# Comparative genomic analysis of 255 *Oenococcus oeni* isolates from China: unveiling strain diversity and genotype-phenotype associations of acid resistance

**DOI:** 10.1128/spectrum.03265-24

**Published:** 2025-04-22

**Authors:** Wei Chi, Hanwen Zhang, Xinyi Li, Yeqin Zhou, Qiang Meng, Ling He, Yafan Yang, Shuwen Liu, Kan Shi

**Affiliations:** 1 College of Enology, College of Horticulture, Shaanxi Engineering Research Center for Viti-Viniculture, Viti-Viniculture Engineering Technology Center of State Forestry and Grassland Administration, Heyang Experimental and Demonstrational Stations for Grape, Ningxia Helan Mountain's East Foothill Wine Experiment and Demonstration Station, Northwest A&F University, Yangling, Shaanxi, China; 2 College of Food Science and Technology, Northwest University, Xi’an, Shaanxi, China; Tianjin University, Tianjin, China

**Keywords:** lactic acid bacteria, pan-genome, genomic island, horizontal gene transfer, genome-wide association study

## Abstract

*Oenococcus oeni,* the only species of lactic acid bacteria capable of fully completing malolactic fermentation under challenging wine conditions, continues to intrigue researchers owing to its remarkable adaptability, particularly in combating acid stress. However, the mechanism underlying its superior adaptation to wine stresses still remains elusive due to the lack of viable genetic manipulation tools for this species. In this study, we conducted genomic sequencing and acid resistance phenotype analysis of 255 *O. oeni* isolates derived from diverse wine regions across China, aiming to elucidate their strain diversity and genotype-phenotype associations of acid resistance through comparative genomics. A significant correlation between phenotypes and evolutionary relationships was observed. Notably, phylogroup B predominantly consisted of acid-resistant isolates, primarily originating from Shandong and Shaanxi wine regions. Furthermore, we uncovered a noteworthy linkage between prophage genomic islands and acid resistance phenotype. Using genome-wide association studies, we identified key genes correlated with acid resistance, primarily involved in carbohydrates and amino acid metabolism processes. This study offers profound insights into the genetic diversity and genetic basis underlying adaptation mechanisms to acid stress in *O. oeni*.

**IMPORTANCE:**

This study provides valuable insights into the genetic basis of acid resistance in *Oenococcus oeni*, a key lactic acid bacterium in winemaking. By analyzing 255 isolates from diverse wine regions in China, we identified significant correlations between strain diversity, genomic islands, and acid resistance phenotypes. Our findings reveal that certain prophage-related genomic islands and specific genes are closely linked to acid resistance, offering a deeper understanding of how *O. oeni* adapts to acidic environments. These discoveries not only advance our knowledge of microbial stress responses but also pave the way for selecting and engineering acid-resistant strains, enhancing malolactic fermentation efficiency and wine quality. This research underscores the importance of genomics in improving winemaking practices and addressing challenges posed by high-acidity wines.

## INTRODUCTION

Malolactic fermentation (MLF) is a critical process in winemaking, particularly red wine. During this process, lactic acid bacteria (LAB) convert the sharp-tasting malic acid into the milder lactic acid, which reduces the wine’s acidity, enhances its flavor and aroma, and improves microbial stability (1). However, winemakers often encounter several challenges in the MLF process, such as prolonged fermentation, incomplete fermentation, and contamination by undesirable microbes (2–4). These challenges mainly arise from the difficulty of LAB to adapt to the harsh conditions of winemaking, including high acidity, high ethanol content, low temperatures, high levels of sulfur dioxide (SO_2_), and nutrient scarcity (5). The growth of LAB is notably inhibited under acidic stress, which can significantly hinder the fermentation process. Consequently, the selection of LAB strains that exhibit strong acid resistance is imperative to ensure the stability and efficiency of MLF. *Oenococcus oeni* is the most commonly employed strain in MLF due to its remarkable adaptability to the stress conditions prevalent in wine. In winemaking, MLF is typically carried out under monoculture conditions by inoculating a selected strain of *O. oeni* (6). Although other LAB species, such as *Lactobacillus*, *Pediococcus*, and *Leuconostoc*, are also capable of performing MLF, they often struggle to survive the extreme conditions in wine, leading to premature fermentation cessation or the production of harmful metabolites, which have a detrimental effect on wine quality (3). Despite *O. oeni*’s ability to tolerate some of these stress factors, its growth can still be inhibited if the wine’s acidity is excessively high (7). It is therefore vital to select *O. oeni* strains with enhanced acid resistance in order to ensure the successful progression of MLF. Such strains can enhance fermentation efficiency, reduce fermentation costs, mitigate the risk of microbial contamination, and ultimately elevate the overall quality of the wine (4, 8).

In contrast to other LAB, there is a paucity of suitable molecular biology techniques to genetically modify *O. oeni*, rendering it challenging to investigate gene function and regulatory mechanisms in *O. oeni* (5). Researchers have devised methods such as electroporation and antisense RNA to research *O. oeni* at the molecular level (9). Nevertheless, these methodologies encounter limited utility due to issues such as poor reproducibility. While heterologous expression in other LAB has partially addressed the difficulty of genetic modification in *O. oeni* (9). Despite these advances, challenges remain, resulting in low gene expression levels and potentially inaccurate outcomes due to differences in cellular environments and metabolic pathways among LAB. In contrast, genomics offers a means of effectively studying the stress response mechanism of *O. oeni* at the molecular level, significantly enhancing research efficiency. By analyzing the genome, we can find potential genotype-phenotype associations, which serve as a solid foundation for future genetic confirmation.

Microorganisms can adapt to stressful environments through horizontal gene transfer (HGT), which results in the generation of a pan-genome comprising multiple accessory genes (10). Comparative genomics analysis of the *O. oeni* pan-genome can reveal evolutionary relationships among strains and elucidate strain diversity associated with phenotypes. Currently, comparative genomics is frequently integrated with genome-wide association studies (GWASs) to more comprehensively elucidate correlations between genotype and phenotype (11). GWAS has been employed to investigate a multitude of statistical associations related to bacterial pathogenicity, antibiotic resistance, and other traits (12).

In this study, we sequenced, assembled, and annotated 255 *O. oeni* isolates for large-scale comparative genomic analysis. Subsequently, the isolates were subjected to an assessment of their resistance to acid stress environments. By integrating comparative genomics with GWAS, we elucidated the evolutionary relationships among *O. oeni* isolates and investigated genotype-phenotype associations of acid resistance. The primary objectives of this study were to examine the relationship between isolation sources, strain diversity, and genomic islands, as well as to identify genes associated with acid resistance. Our comprehensive analysis of population structural characteristics provides novel insights into the genetic traits of *O. oeni* and offers a foundation for further investigation into the potential application of acid-resistant *O. oeni* in the winemaking industry.

## RESULTS

### Genomic and pan-genomic characterization

**Fig 1:**
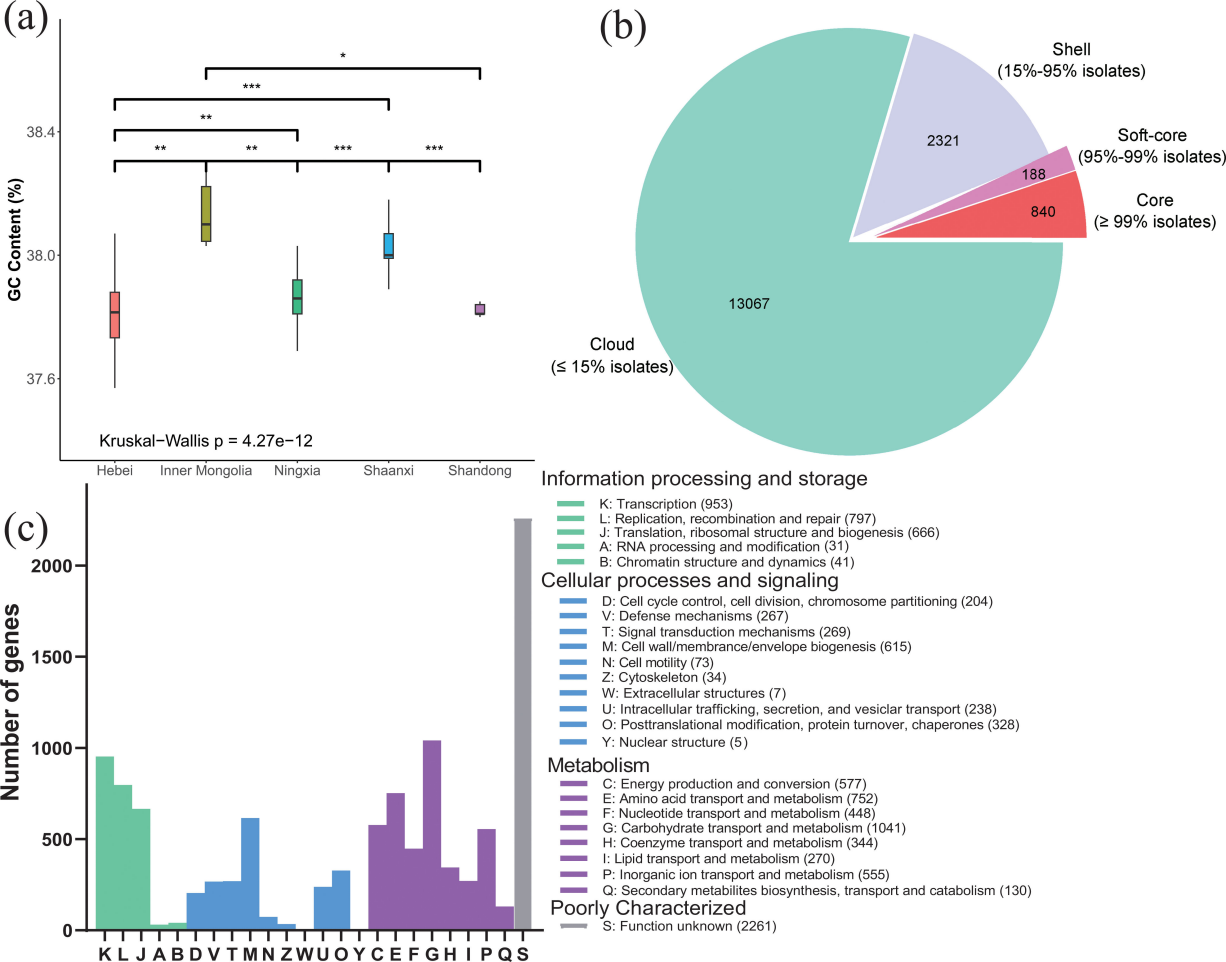
Genomic characterization and functional annotation of *O. oeni*. (a) Boxplot illustrating the GC content (%) of *O. oeni* genomes across various provinces of China. Significant differences in GC content were observed between isolates from different provinces (*P* < 0.05, Kruskal-Wallis test). Pairwise comparisons indicated the following significance levels: *P < 0.05; **P < 0.01; ***P < 0.001 (Wilcoxon tests). Only significant results are presented in the figure. (b) Fan chart depicting the distribution of pan-genomic genes among 255 *O. oeni* isolates. (c) Functional annotation of the pan-genome utilizing the COG database. Pan-genome genes are categorized into four groups: information processing and storage, cellular processing and signaling, metabolism, and poorly characterized.

In order to gain a comprehensive understanding of the strain diversity of *O. oeni* comprehensively, we utilized 255 *O. oeni* isolates from various regions of China between 1999 and 2016 (Table S1). Among these, seven isolates were obtained by downloading from the NCBI database. The genomes of the remaining 248 isolates were sequenced and assembled in this study. The average genome size of 255 isolates was 2.1 Mb, with an average GC content of 37.88%, an average N50 of 1.12 Mb, and an average depth of 912× (Table S1). Based on the genomic data of 243 *O.oeni* uploaded to the NCBI database, the genome size ranges from 1.7 to 2.5 Mb, with an N50 ranging from 4.7 kb to 2.0 Mb. The GC content is between 37.5% and 38.5%. The sequencing coverage of the bacterial genomes typically ranges from 30× to 150×, with coverage exceeding 150× can enhance the accuracy of genome data (13). Our genomic data fall within these reasonable ranges. The GC content of *O. oeni* exhibited significant regional differences (*P* = 4.27e−12, Kruskal-Wallis test) (Fig. 1a).

After pan-genomic analysis of 255 *O. oeni* isolates, a total of 16,416 pan-genes were identified. Among these, the core genome comprised 1,028 genes, including 840 core genes and 188 soft-core genes (Fig. 1b). The soft-core genes are those present in 95–99% of the isolates, while the core genes are those found in more than 99% of the isolates. The pan-genome fit curve exhibited an increasing trend. The fit of Heaps' law with an exponent of *γ* = 0.37 indicated that the pan-genome of *O. oeni* was open (Fig. S1). The exponent *γ* of the cumulative curve of the core genome was close to 0, indicating that the core genome was in a stable state. Among the 10,906 pan-genes annotated by the COG database, except for genes of unknown function, the majority are involved in metabolism, cellular processes, and signaling, as well as information processing and storage (Fig. 1c).

### Analysis of the strain diversity

Pairwise average nucleotide identity (ANI) is widely used in genomic studies as a reliable metric for determining species boundaries. An ANI value exceeding 95% generally suggests that the organisms belong to the same species (14). The PSU-1 strain (assembly accession NC_008528.1) was the first *O. oeni* strain to have its whole-genome sequenced. Over the years, it has been extensively studied, with high-quality data and comprehensive annotations, making it a widely used reference genome in *O. oeni* research. In this study, we used PSU-1 as the reference strain and compared it with all the isolates. The ANI values ranged from 98.88% to 99.85%, all exceeding 95%, thereby confirming that the isolates all belong to *O. oeni*.

**Fig 2:**
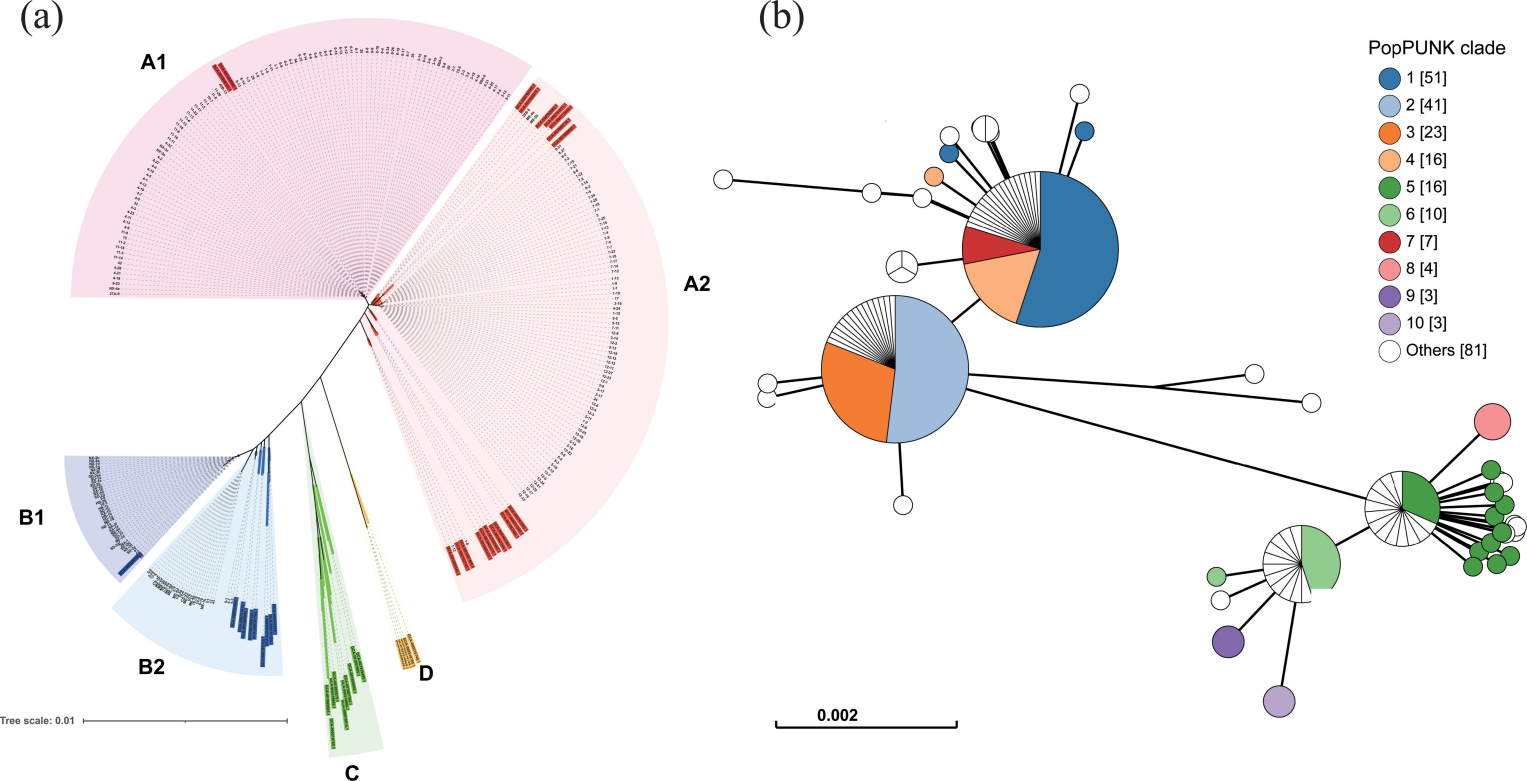
Strain diversity analysis of *O. oeni* using diverse methodologies. (a) Phylogenetic tree constructed from 300 strains, including 45 strains (highlighted with shaded labels) randomly selected from the data set of Lorentzen et al. 1, representing all four phylogroups. The strains shaded in red, blue, green, and gold correspond to phylogroups A, B, C, and D, respectively. The 255 isolates analyzed in this study are classified into either phylogroup A or B. (b) Results of PopPUNK analysis based on genome-wide variable length k-mers.

Lorentzen et al. 1 classified *O. oeni* strains from wine, cider, and kombucha into four phylogroups (A, B, C, and D) based on the core genome phylogenetic tree. To assign the 255 *O. oeni* isolates used in this study to these phylogroups, we randomly selected 45 strains from the data set of Lorentzen et al. (Table S2), which represent all four phylogroups. These strains served as references to construct a phylogenetic tree alongside the 255 isolates from this study, allowing us to determine the phylogroups of our isolates. The unrooted phylogenetic tree based on the core genome showed that these isolates belong to phylogroups A and B. They were further subdivided into four subgroups A1, A2, B1, and B2 (Fig. 2a).

Strain diversity analysis helps in understanding evolutionary relationships and is crucial for comprehending bacterial adaptation to diverse environments. Multilocus sequence typing (MLST) is a standard method for analyzing strain diversity, with a well-established and widely accepted analytical workflow. The results obtained through MLST are recognized globally. It classifies all strains into different sequence types (STs), each ST is represented by “ST” followed by a number. Among the 255 isolates in this study, 53 different STs were identified (Table S1).

Since MLST is based on only seven housekeeping genes, it may struggle to accurately resolve complex strain diversity. In contrast, the PopPUNK method relies on whole-genome variations for strain diversity analysis, offering a higher resolution compared to MLST. We represented the clades obtained from the PopPUNK as “clade” followed by a number. The PopPUNK analysis yielded 91 clades (Table S1). Among these, clades 1 and 2 contained the highest number of isolates, collectively representing 36.1% (92/255) of the total (Fig. 2b).

### Effect of acidic stress on *O. oeni* survival and classification of acid resistance phenotype

The experimental group was set to pH 3.4 to investigate the effect of an acidic environment on *O. oeni* survival, while the control group was maintained at pH 4.8, the optimal pH for its growth. After 7 days of incubation at 26°C, colony counts were performed. Colony counts were significantly lower in the experimental group (2.2 × 10^8^ CFU/mL) compared to the control group (6.5 × 10^8^ CFU/mL, *P* < 0.001; Fig. S2a). Isolates were classified as resistant or sensitive based on whether their survival rate was above or below the median (10.5%), respectively. Resistant isolates showed a significantly higher survival rate (*P* < 0.001; Fig. S2b) and GC content (*P* < 0.001; Fig. S2c).

### Correlation analysis between strain diversity and acid resistance phenotypes

**Fig 3:**
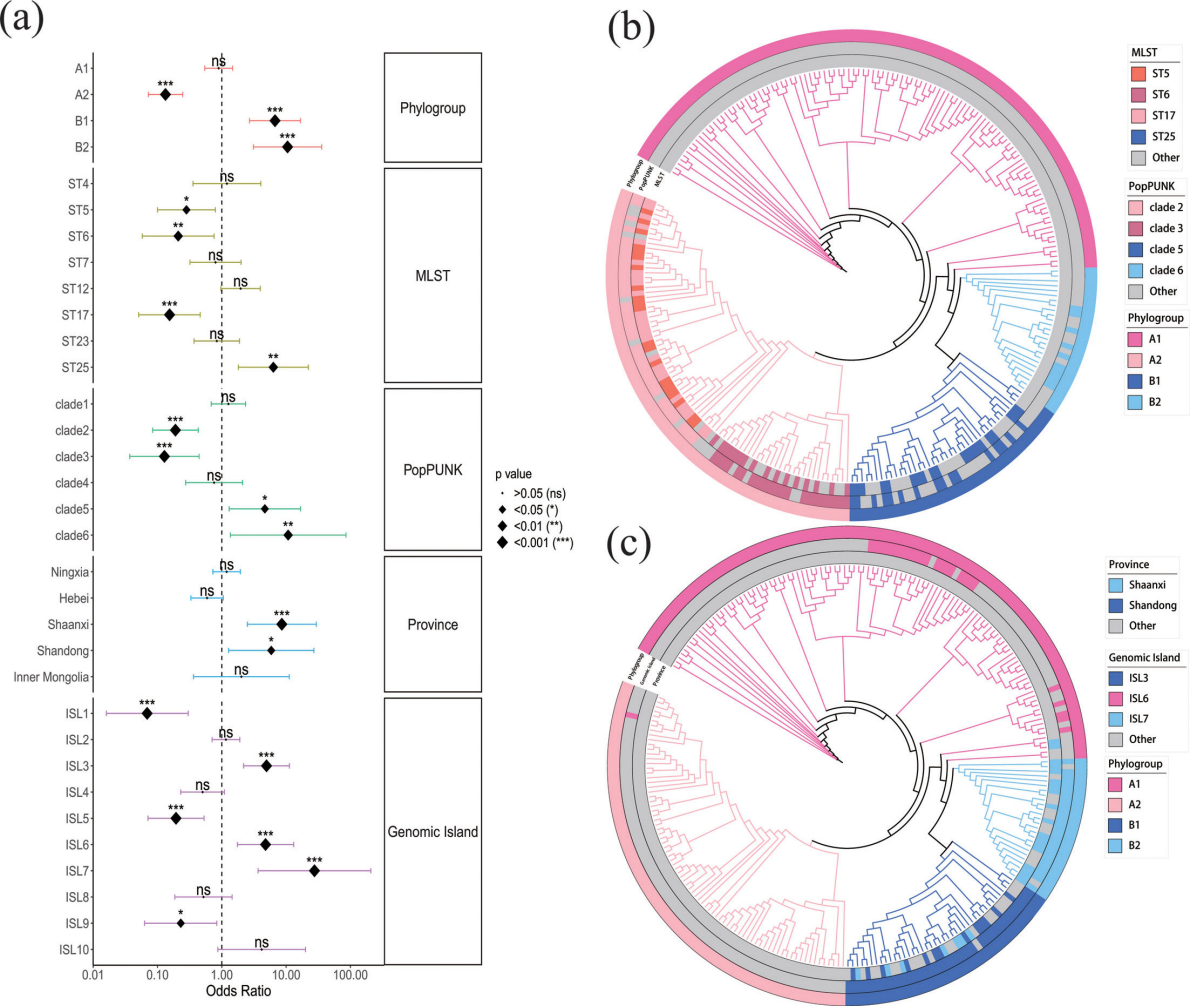
(a) Forest plot showing the correlation between acid resistance phenotype and variables such as strain diversity, isolation regions, and genomic islands. The points represent the odds ratio (OR) of each variable, with horizontal lines indicating the 95% confidence intervals; the vertical dashed line (*x* = 1) indicates no correlation, and the deviation of OR from 1 reflects the strength of the association (OR > 1 for positive correlation, OR < 1 for negative correlation). Statistical significance is indicated as follows: ns, *P* > 0.05; *P < 0.05; **P < 0.01; ***P < 0.001 (chi-square tests). (b) Distribution of MLST and PopPUNK results within phylogroups. Concentric rings from innermost to outermost represent MLST, PopPUNK, and phylogroups. (c) Distribution of isolation regions and genomic islands within phylogroups. Concentric rings from innermost to outermost represent isolation regions, genomic islands, and phylogroups.

To identify strain diversity associated with acid resistance phenotype, we analyzed the correlation between strain diversity and phenotype. Chi-square testing revealed that phylogroups B1 and B2 were significantly positively correlated with acid resistance (*P* < 0.001, odds ratio [OR] > 1), while phylogroup A2 showed a significant negative correlation (*P* < 0.001, OR < 1) (Fig. 3a).

From the 53 STs identified through MLST analysis, 8 major STs, each comprising more than 10 isolates, were selected for further study (Table S3). Chi-square testing revealed a significant association between four STs (ST5, ST6, ST17, and ST25) and acid resistance phenotype (*P* < 0.05) (Fig. 3a). An OR >1 indicates a positive association, while OR < 1 suggests a negative association. In this context, a positive correlation denotes a predominance of acid-resistant isolates. Notably, only ST25 showed a positive correlation with acid resistance (OR > 1), while the other three STs were negatively correlated (OR < 1) (Fig. 3a). ST25 belongs to phylogroup B1 (Fig. 3b).

Among the 91 clades identified through the PopPUNK analysis, 6 major clades, each comprising more than 10 isolates, were selected for further study (Table S3). Clades 2, 3, 5, and 6 were significantly correlated with acid resistance phenotype (*P* < 0.05) (Fig. 3a). Clades 5 and 6 showed positive correlations with resistance (OR >1), while clades 2 and 3 exhibited negative correlations (OR <1) (Fig. 3a). Clades 5 and 6 belong to phylogroups B1 and B2, respectively (Fig. 3b).

Based on the above results, acid-resistant *O.oeni* isolates are correlated with strain diversity, primarily associated with ST25 from MLST analysis, as well as clades 5 and 6 identified through PopPUNK analysis. These strain diversities belong to phylogroups B1 and B2. Therefore, selecting isolates from phylogroups B1 and B2 may enhance the likelihood of obtaining acid-resistant isolates.

### Correlation analysis between isolation regions, genomic islands, and phenotypes

Genomic islands are defined as genome regions that are enriched for specific functional genes, which may be closely linked to the phenotype traits (15). After identifying the genomic islands of the isolates using IslandViewer 4 (16), their similarities were calculated with IslandCompare (17), and clustering algorithms grouped them into clusters. These clusters were named with the prefix “ISL” followed by a number. In this study, we identified 10 predominant genomic islands, each comprising more than 15 isolates (Table S3). Six of these showed a significant correlation with acid resistance phenotype, including ISL1, ISL3, ISL5, ISL6, ISL7, and ISL9 (Fig. 3a). Among them, ISL3, ISL6, and ISL7 were positively correlated with acid resistance (OR > 1), while ISL1, ISL5, and ISL9 were negatively correlated with the acid resistance phenotype (OR < 1) (Fig. 3a).

**Fig 4:**
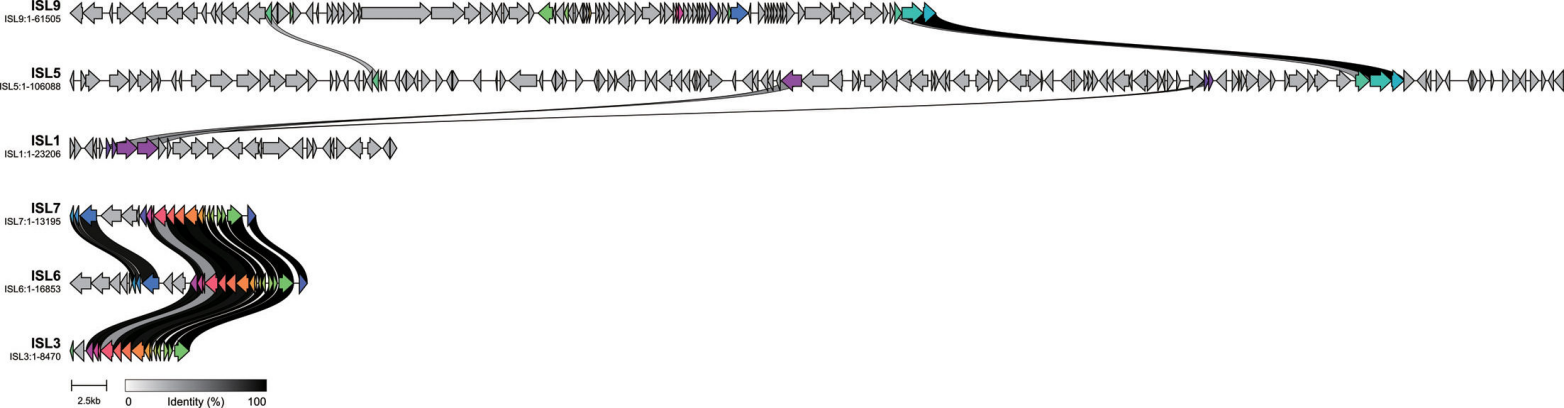
Synteny analysis of genomic islands. The lines connecting the different genomic islands represent homologous genes, with the color of the lines indicating the level of similarity between the homologous genomes. The numbers below the genomic island names represent the length of each genomic island.

PHASTER (18) revealed that ISL3, ISL6, and ISL7 were encoded prophage genes. BLAST (19) sequence alignment showed high identities with phage phiS11 (96.46%, 99.99%, and 98.58%, respectively), confirming their phage origin. In contrast, ISL1, ISL5, and ISL9, which were negatively correlated with the resistant phenotype (OR < 1), did not align with phage sequences. Synteny analysis showed that prophage genomic islands contain a higher number of homologous genes, while non-prophage genomic islands have fewer homologs. No homologous genes were identified between the prophage and non-prophage genomic islands (Fig. 4). Functional annotation of these genomic islands is shown in Table S4. Phylogroups B1 and B2, significantly associated with acid resistance, primarily contain ISL3 and ISL7, respectively (Fig. 3c). These findings suggest that exogenous genes acquired via phage transduction may play an important role in influencing the acid resistance phenotype of *O. oeni*.

The isolation region refers to the provinces in China where the *O. oeni* isolates were obtained. Analysis of isolation regions revealed that isolates from Shaanxi and Shandong provinces were significantly positively correlated with acid resistance phenotype (*P* < 0.05, OR > 1) (Fig. 3a). All isolates from Shandong province belonged to phylogroup B1, while most isolates from Shaanxi province (*n* = 16, 64%) belonged to phylogroup B2 (Fig. 3c). Therefore, isolates from these two regions predominantly belong to phylogroups B1 and B2, suggesting that screening *O. oeni* strains from these regions may facilitate the acquisition of acid resistance strains.

### Genome-wide association studies identified genes associated with acid resistance

**Fig 5:**
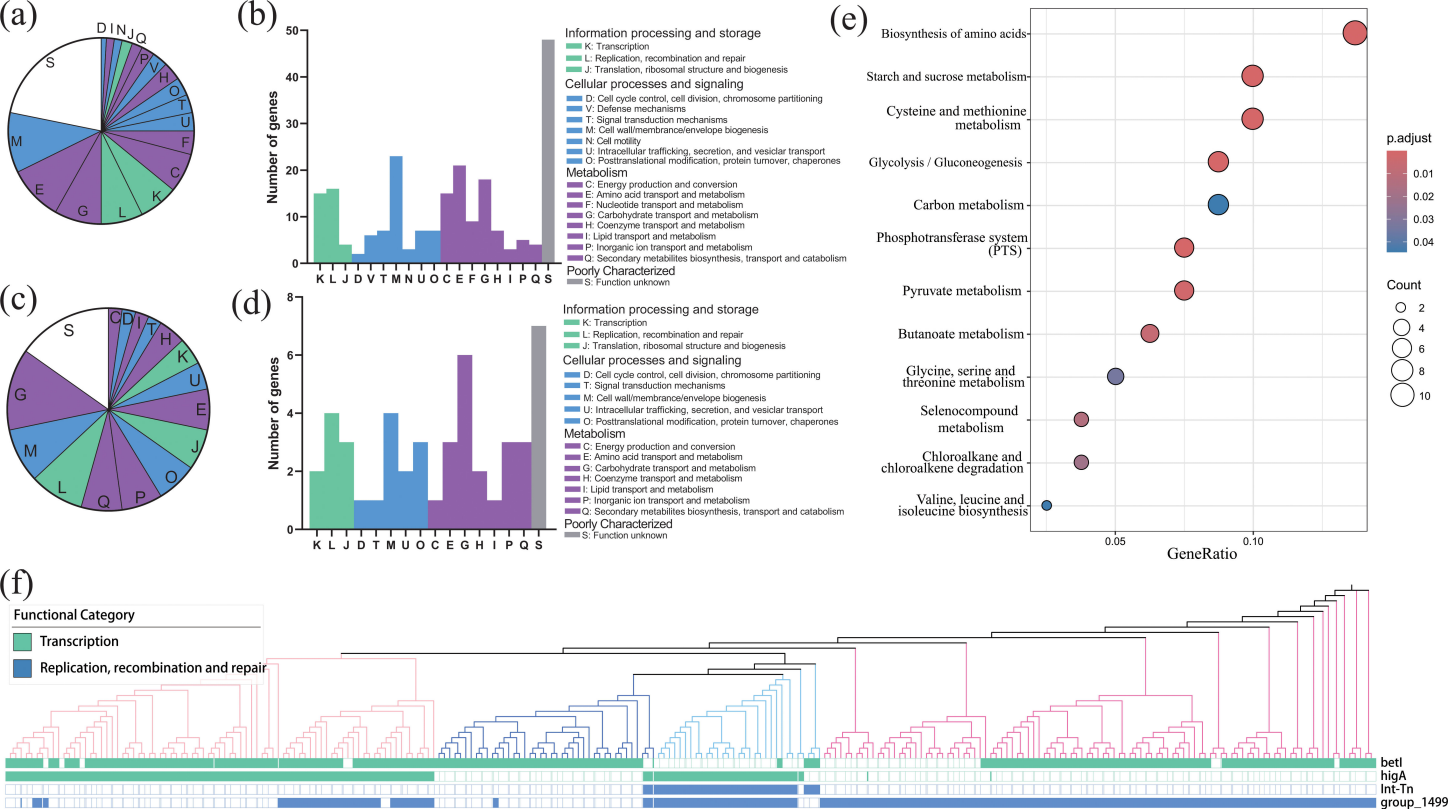
Functional annotation results of genes associated with acid resistance identified by GWAS. (a) Fan chart and (b) bar graph displaying COG annotations for genes in phylogroup A. (c) Fan chart and (d) bar graph illustrating COG annotations for genes in phylogroup B. (e) KEGG pathway enrichment analysis of candidate genes from phylogroups A and B presented in a dot plot. The *x*-axis represents the gene ratio (the ratio of genes in a pathway to the total number of genes analyzed), and the *y*-axis lists the significantly enriched KEGG pathways. The size of the dots corresponds to the number of genes involved in each pathway, and the color reflects the level of statistical significance. (f) Genes significantly associated with acid resistance are shared within phylogroups A and B. The presence or absence of genes within different isolates is indicated by solid and hollow symbols, respectively. The different colors of branches represent the different phylogroups, from left to right: phylogroups A2, B1, B2, and A1.

In order to elucidate the genetic basis underlying adaptation to acid stress, we conducted GWAS with the objective of establishing the relationship between genetic variation and acid resistance phenotypes. It is possible that genetic variation associated with acid resistance may diverge across different phylogroups due to discrepancies in living environmental conditions. This has prompted us to conduct separate GWAS for phylogroups A and B. The Q-Q plots are drawn in Fig. S3. In phylogroup A, 218 candidate genes were found to be significantly associated with the phenotype (Table S5, Fig. S4), which were categorized into 18 functional categories using the COG database. The genes were primarily involved in cell wall/membrane/envelope biosynthesis (*n* = 23, 10.6%), amino acid transport and metabolism (*n* = 21, 9.6%), and carbohydrate transport and metabolism (*n* = 18, 8.3%) (Fig. 5a and b). Meanwhile, phylogroup B exhibited 43 identified candidate genes (Table S6, Fig. S4), distributed among 16 functional categories. These were predominantly associated with carbohydrate transport and metabolism (*n* = 6, 14.0%), cell wall/membrane/envelope biosynthesis (*n* = 4, 9.3%), as well as replication, recombination, and repair (*n* = 4, 9.3%), (Fig. 5c and d).

We performed pathway enrichment analysis on genes identified in phylogroups A and B. These genes are primarily involved in biosynthesis of amino acids, starch and sucrose metabolism, cysteine and methionine metabolism, and glycolysis/gluconeogenesis (Fig. 5e). Therefore, genes associated with acid resistance are mainly linked to carbohydrates and amino acid metabolism. Four candidate genes (*betI*, *higA*, *Int-Tn*, and *group_1499*) were significantly present in both phylogroups (Fig. S4). *betI* and *higA* are transcriptional regulators, *Int-Tn* is a bacteriophage integrase, and group_1499 encodes a nuclease. These genes are involved in replication, recombination, and repair, as well as transcription (Fig. 5f).

## DISCUSSION

Strain diversity and acid resistance phenotype correlation analysis revealed a significant association between phylogroups B1 and B2 with the acid resistance phenotype. Isolates from these two phylogroups are primarily from the Shandong and Shaanxi provinces, and most contain genomic islands associated with the bacteriophage. GWAS further identified that genes related to acid resistance phenotype are primarily involved in carbohydrate and amino acid metabolism. We identified four genes associated with the acid resistance phenotype.

Isolates from Hebei were primarily acid-sensitive while those from Shandong and Shaanxi provinces were predominantly acid-resistant. This may be linked to the acidic conditions in the wine environments where these strains thrive. During grape maturation, sugar content increases and acidity decreases. Grape ripening is influenced by various factors, including agricultural practices and climatic conditions such as temperature, precipitation, and sunlight. Temperature is a critical factor in grape maturation. Higher temperatures accelerate ripening, allowing more time for sugar accumulation and reducing organic acid content (20). The climates of Shandong and Hebei are similar, exhibiting characteristics of temperate monsoon climates (21). However, the isolates were collected in different years, and grape ripening has accelerated over time due to global warming. As a result, the acidity of Hebei grapes in 2016 was lower than that of Shandong grapes in 1999. Grapes rely heavily on sunlight for photosynthesis during ripening, and insufficient sunlight caused by rainy weather can hinder this process (22). In Shaanxi, autumn is characterized by high humidity, with 52% of the annual rainfall during the grape ripening period (23). Excessive rainfall reduces sunlight and affects grape maturation. Agricultural practices also significantly influence grape acidity in Shaanxi. Excessive rainfall during the ripening period creates favorable conditions for fungal growth, leading to grape rot. To minimize losses, early harvests are often employed (24). Early harvesting results in lower grape ripeness and higher acidity in wines. Therefore, isolates from Shandong and Shaanxi, which are selected from wines made with less ripe grapes, show strong acid resistance.

HGT refers to the process by which microorganisms acquire genetic fragments from the environment or other organisms, integrating them into their own genomes. This transfer occurs through three main mechanisms: transformation, where DNA fragments are absorbed from the environment; transduction, where bacteriophages mediate the transfer; and conjugation, which is mediated by plasmids or transposons. HGT is increasingly recognized as a driving force in bacterial evolution (25), providing bacteria with a survival advantage in specific environments. Under the influence of natural selection, genes that are harmful or unnecessary are typically lost, while beneficial foreign genes can become fixed within the genome through natural selection (26). Therefore, retained foreign genes are often associated with adaptability. An important outcome of HGT is the formation of genomic islands, which are large regions of foreign genes that may include those associated with acid resistance, antibiotic resistance, and salt tolerance (27). These genes are often tightly linked to the bacterium’s survival advantage under specific environmental stresses. We found that the genomic islands associated with acid resistance are related to prophage genes, which are typically acquired through transduction, suggesting that phage-related foreign genes may play a crucial role in the acid resistance of *O. oeni*.

Gene enrichment analysis revealed that pathways significantly associated with the acid resistance phenotype are primarily related to carbohydrate and amino acid metabolism. Bacteria require energy to resist acid stress, such as using proton pumps to expel protons from the cell (28). These metabolic processes provide energy that enhances the bacterium’s ability to withstand acid stress (29). The four genes identified in this study were identified through GWAS. The *betI* gene is a transcriptional regulator associated with stress resistance, has been shown to play a role in antibiotic resistance (30) and osmoregulatory stress (31). Together with the *betAB* genes, *betI* forms an operon that regulates intracellular choline and glycine betaine levels (32, 33), which may neutralize H^+^ ions and contribute to acid resistance. Choline also helps maintain membrane stability under acidic conditions, enhancing bacterial adaptability (34). The *higA* gene, another transcriptional regulator, belongs to the MqsA family and enhances bacterial resistance to toxins (35) and bile acids (36). However, its role in acid resistance remains uncertain and requires further investigation. The *Int-Tn* gene encodes a phage integrase involved in transduction, facilitating the integration of prophage genes into the *O. oeni* genome. These foreign genes may contribute to acid stress adaptation. Group_1499 is a member of the RusA family and functions as a bacterial nucleic acid endonuclease involved in DNA repair (37). Repairing DNA and protein damage is a key bacterial response to acid stress (38). This gene may contribute to stress resistance by repairing acid-induced genetic damage.

In this study, we established a link between the genetic background and acid resistance phenotype by analyzing the strain diversity, providing a theoretical foundation for the screening and identification of acid-resistant strains. We identified key pathways and genes associated with acid resistance, contributing to further understanding of the underlying mechanisms. Future studies could use omics or gene editing approaches to explore the acid resistance mechanisms of *O. oeni* in greater detail.

## MATERIALS AND METHODS

### Genomic DNA extraction

In this study, we utilized 255 isolates of *O. oeni* collected from various wine-producing regions in China between 1999 and 2016. These isolates were obtained from a previous screening by our research group, aiming to ensure an adequate sample size while covering a wide range of regions to investigate the potential correlation between the isolation source and acid resistance phenotype. Seven isolates had previously undergone genome sequencing, with genomic data obtained from the NCBI database. These included SD-2a (NZ_CP087569.1), a1 (GCF_004377635.1), a3 (GCF_004377595.1), b1 (GCF_004377535.1), b2 (GCF_004377585.1), c1 (GCF_004377555.1), and c2 (GCF_004377505.1). Genomic DNA extraction was conducted on the remaining 248 isolates using the D3350 Bacterial DNA Kit according to the manufacturer’s instructions from OMEGA Bio-Tek Co. (Norcross, GA, USA). Prior to sequencing, the quality of the DNA was evaluated using 1% agarose gel electrophoresis.

### Whole-genome sequencing and assembly

A total of 248 isolates were subjected to whole-genome sequencing using Illumina PE150 high-throughput sequencing technology, generating paired-end 150 bp reads. Approximately 2 Gb of raw data were obtained for each isolate. Trimmomatic v0.39 (39) was employed for noise reduction and adaptor removal. Reads with an average quality score below 20 or a length shorter than 36 bp were discarded. Quality control of raw data were performed using FastQC v0.11.9 (40), and the results were then integrated using MultiQC v1.11 (41) to visualize the quality of the noise-reduced reads. The reads, with adaptor removed, were assembled using SPAdes v3.15.4 (42), with the “--careful” parameter enabled. This parameter enables more stringent error correction methods, significantly improving the accuracy of the assembly results. The complete genome of the PSU-1 strain was used as the reference genome for the assembly. The sequence length, N50, and GC content were assessed using QUAST v5.0.2 (43) with default parameters.

### Growth conditions and phenotype testing

The isolates were incubated in liquid medium with pH 4.8 comprising the following components: 13.6 g/L fructose, 27 g/L glucose, 10 g/L yeast extraction, 10 g/L yeast peptone, 3.3 g/L l-malic acid, 0.3 g/L l-Cysteine hydrochloride anhydrous, 0.2 g/L MgSO_4_·7 H_2_O, and 0.05 g/L MnSO_4_·4 H_2_O. Incubation was conducted at 26°C until the optical density at 600 nm (OD_600_) reached 0.8. Subsequently, the bacterial suspensions were inoculated into liquid media with different pH values, categorized into experimental (pH 3.4) and control (pH 4.8) groups, and incubated for 120 h at 26°C. The incubated suspension was plated on agar plates at pH 4.8, followed by incubation at 26°C for 7 days. Colony counts were conducted for both the experimental and control groups. The survival rate was calculated using the following formula: Survival rate (%) = (colony count in the experimental group/colony count in the control group) × 100%. The classification of resistance and sensitivity was based on the median survival rate.

### Strain diversity and pan-genomic analyses

ANI between strains was assessed using FastANI v1.33 (44). The strain diversity of *O. oeni* was analyzed using PopPUNK v2.6.3 (45), which is based on variable-length k-mers. MLST was conducted using mlst v2.23.0 (46), in accordance with the typing schemes proposed by Bridier (47). Genomic islands were analyzed and compared using IslandViewer 4 (16) and IslandCompare (17). Synteny analysis of genomic islands was conducted using Clinker v0.0.31 (48). Following genome annotation using Prokka v1.12 (49), pan-genome analysis was conducted using Roary v3.13.0 (50). Phylogenetic trees were generated using FastTree v2.1.11 (51) to delineate the relationships between different strains.

### Correlation analysis

Correlation analysis was conducted using IBM SPSS Statistics 23, employing chi-square tests and OR analysis to investigate the relationship between different strain diversity and phenotypes. The *P* value is used to determine the statistical significance of the results, while the OR quantifies the strength and direction of the association between the two variables. When OR >1, it indicates a positive correlation with the phenotype, whereas OR < 1 suggests a negative correlation. The farther the absolute value of OR is from 1, the stronger the association between the two variables; conversely, an OR closer to 1 indicates a negligible or no association.

### GWASs

GWASs were conducted to examine the genes linked with acid resistance in *O. oeni*. We utilized two types of variant data: variable-length k-mers and gene presence/absence (GPA) data to capture the effects of small-scale variations and complete gene losses on the phenotype. For each variant type, we applied two GWAS models: the linear mixed model (LMM) and the fixed-effects model (52) to enhance the accuracy of the association analysis. Genes significantly associated with the acid resistance phenotype across different variant types and models (i.e., k-mers + LMM, k-mers + fixed-effects, GPA + LMM, and GPA + fixed-effects) were defined as candidate genes.

The assembled genome was fragmented into k-mers ranging from 9 to 100 base pairs in length using FSM-lite (https://github.com/nvalimak/fsm-lite). The gene presence/absence data were generated using Roary v3.13.0 (50). Pyseer v1.3.10 (53) was employed to analyze the association between genes and phenotypes. K-mers were mapped to the complete genome of *O. oeni* PSU-1 using the fastmap algorithm in Bwa (53, 54). The functional annotation of genes was performed using eggNOG-mapper v2.1.4 (55).

## Data Availability

Genomic data for the 248 *O. oeni* isolates have been deposited in the NCBI database under BioProject PRJNA1088411.

## SUPPLEMENTAL MATERIAL

Supplemental figures spectrum.03265-24-s0001.docx Fig. S1 to S4.

Supplemental tables spectrum.03265-24-s0002.xlsx Tables S1 to S6.

ASM does not own the copyrights to Supplemental Material that may be linked to, or accessed through, an article. The authors have granted ASM a non-exclusive, world-wide license to publish the Supplemental Material files. Please contact the corresponding author directly for reuse.
