# Supplementary material for: Comparative genomic analysis of 255 Oenococcus oeni isolates from China: unveiling strain diversity and genotype-phenotype associations of acid resistance
Source: Microbiol Spectr. 2025 Apr 22;13(6):e03265-24. doi: 10.1128/spectrum.03265-24 (PMC12131864; doi:10.1128/spectrum.03265-24)
Supplement: Supplemental figures — Fig. S1 to S4. [file spectrum.03265-24-s0001.docx]

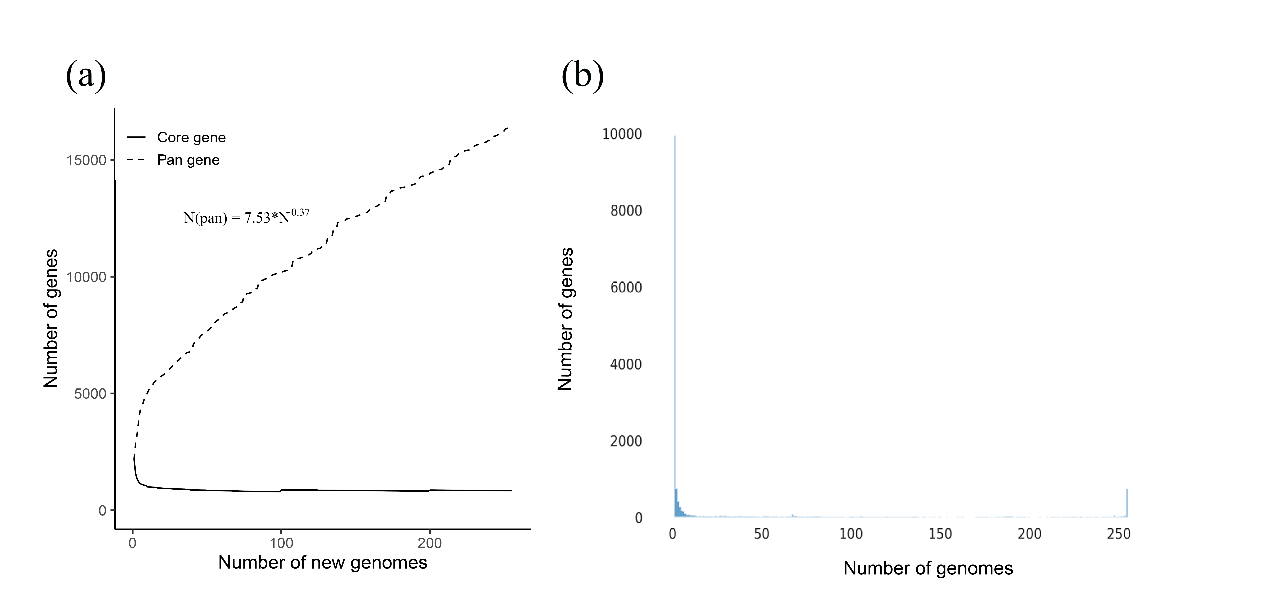


**Fig. S1** Pangenome structure of the 255 *Oenococcus oeni* isolates used in this study. (a) Pangenome accumulation curve showing the number of conserved genes and total genes in the pangenome as a new genome is randomly added. (b) The frequencies of genes in the *O. oeni* pangenome. Bars show the population-level sums of genes for the number of genomes.


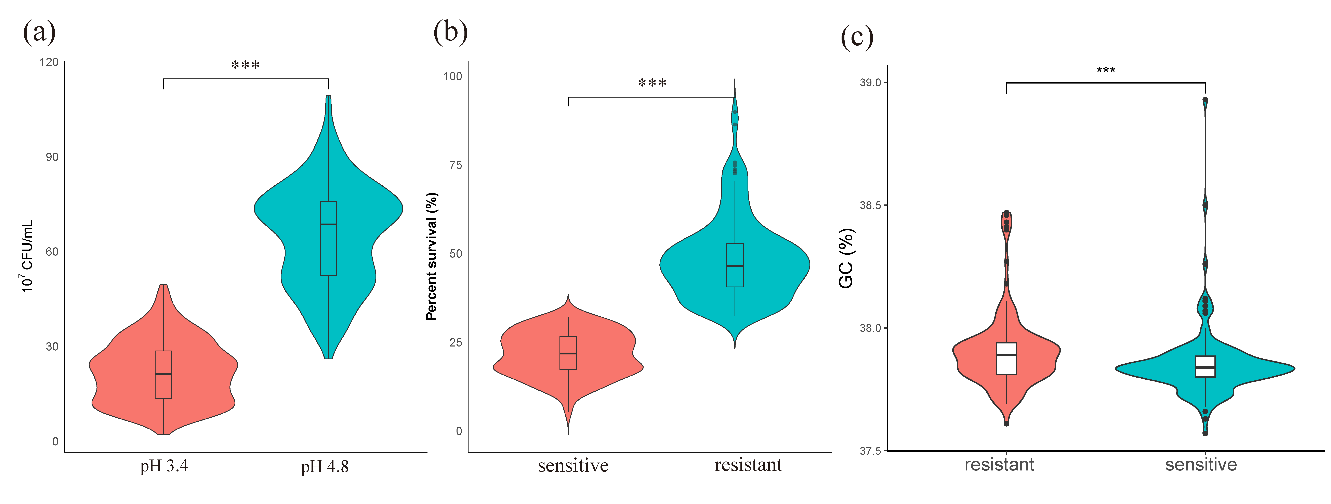


**Fig. S2** Violin polt depicting (a) growth performance of *O. oeni* under acid-stressed (pH 3.4) and optimal (pH 4.8) conditions. Significant differences were observed in (b) survival rate and (c) GC content between acid-resistant and acid-sensitive isolates (*** p < 0.001, Wilcoxon test).


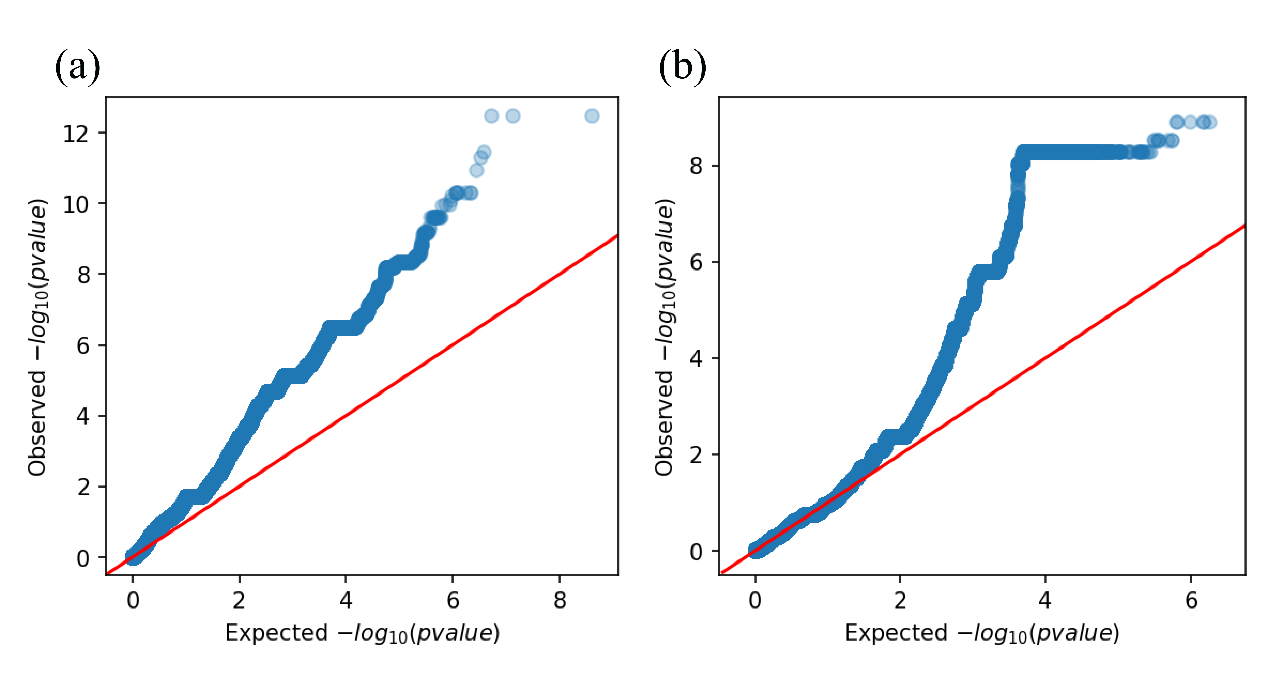


**Fig. S3** Q-Q plot of (a) phylogroup A and (b) phylogroup B isolates associated with acid resistance.


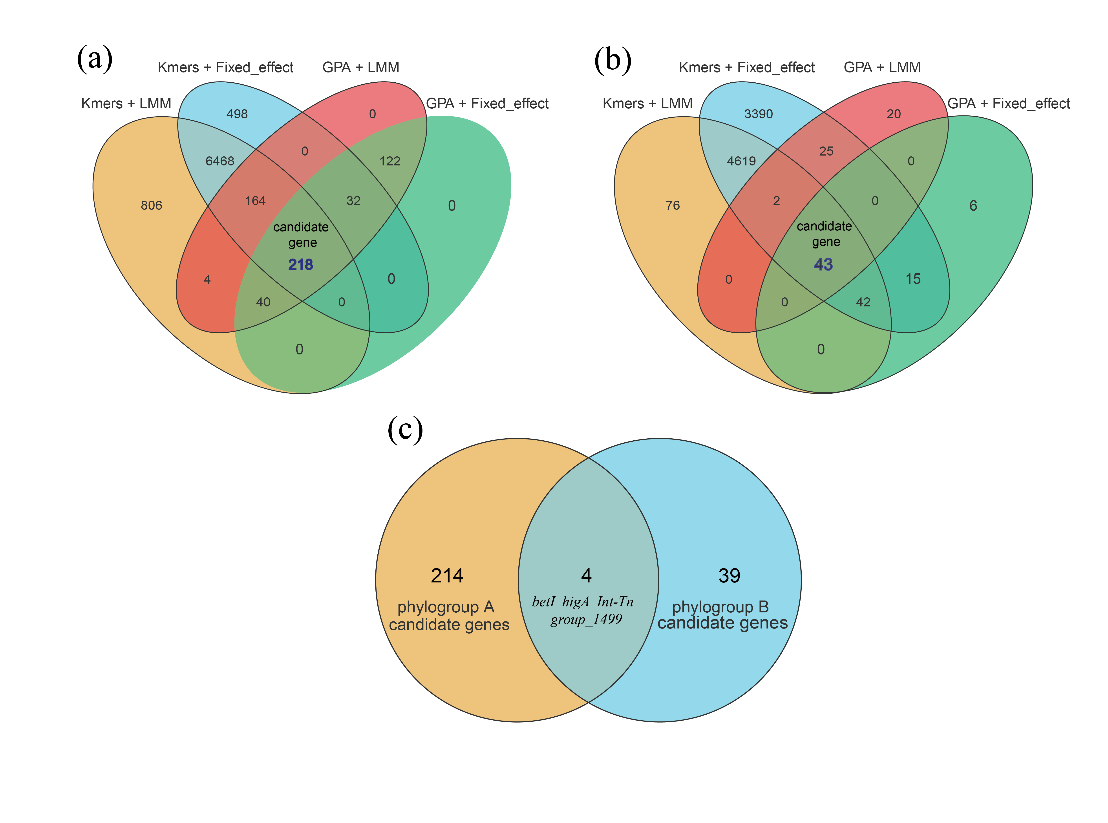


**Fig. S4** Candidate genes associated with phenotypes, identified using two types of genetic variation: K-mers and gene presence/absence (GPA), and analyzed by both linear mixed models (LMM) and fixed-effects models. Genes significantly associated with phenotypes in all variation types and models were defined as candidate genes. (a) Venn diagram of candidate genes associated with phenotypes in phylogroup A. (b) Venn diagram of candidate genes associated with phenotypes in phylogroup B. (c) Venn diagram of genes significantly associated with acid tolerance phenotype in both phylogroups A and B.
